# Using Omics Approaches in the Discovery of Biomarkers for Early Diagnosis of Johne’s Disease in Sheep and Goats

**DOI:** 10.3390/ani11071912

**Published:** 2021-06-27

**Authors:** Palazzo Fiorentina, Camillo Martino, Ylenia Mancini, Maria Grazia De Iorio, John L. Williams, Giulietta Minozzi

**Affiliations:** 1 Faculty of BioScience and Technology for Food, Agriculture and Environment, University of Teramo, 64100 Teramo, Italy; fiorentina.palazzo@sinte.net (P.F.); yleniamanc@gmail.com (Y.M.); 2 Istituto Zooprofilattico Sperimentale dell’Abruzzo e del Molise “G. Caporale” Via Campo Boario, 64100 Teramo, Italy; c.martino@izs.it; 3 Department of Veterinary Medicine DIMEVET, University of Milan, 20133 Milan, Italy; maria.deiorio@unimi.it; 4 School of Animal and Veterinary Sciences, Faculty of Sciences, University of Adelaide, Roseworthy, SA 5371, Australia; john.williams01@adelaide.edu.au; 5 Dipartimento di Scienze Animali, della Nutrizione e degli Alimenti, Università Cattolica del Sacro Cuore, 29122 Piacenza, Italy

**Keywords:** ovine, caprine, paratuberculosis, immunology, diagnostics

## Abstract

**Simple Summary:**

Johne’s disease (JD) is caused by *Mycobacterium avium subsp. paratuberculosis* (MAP) and is an important and emerging problem in livestock. Most JD research has been carried out on cattle, but interest in the pathogenesis and diagnosis of this disease in sheep and goats is greatest in developing countries. Sheep and goats are also a relevant part of livestock production in Europe and Australia, and these species provide an excellent resource to study and better understand the mechanism of survival of MAP and gain insights into possible approaches to control this disease. This review gives an overview of the literature on paratuberculosis in sheep and goats, highlighting the immunological aspects and the potential for “omics” approaches to identify effective biomarkers for the early detection of infection.

**Abstract:**

Johne’s disease (JD) is caused by *Mycobacterium avium subsp. paratuberculosis* (MAP) and is an important and emerging problem in livestock; therefore, its control and prevention is a priority to reduce economic losses and health risks. Most JD research has been carried out on cattle, but interest in the pathogenesis and diagnosis of this disease in sheep and goats is greatest in developing countries. Sheep and goats are also a relevant part of livestock production in Europe and Australia, and these species provide an excellent resource to study and better understand the mechanism of survival of MAP and gain insights into possible approaches to control this disease. This review gives an overview of the literature on paratuberculosis in sheep and goats, highlighting the immunological aspects and the potential for “omics” approaches to identify effective biomarkers for the early detection of infection. As JD has a long incubation period before the disease becomes evident, early diagnosis is important to control the spread of the disease.

## 1. Introduction

**Figure 1:**
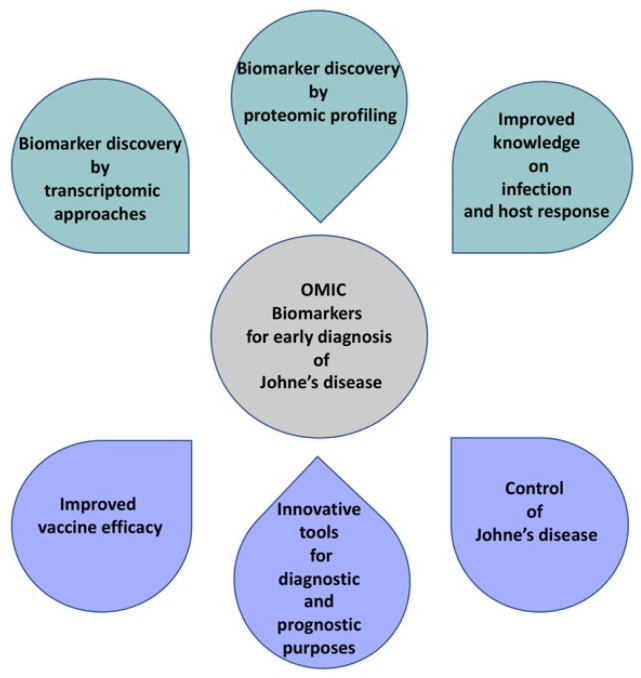
Tools used to identify “omics” biomarkers for early detection of Johne’s disease and their possible applications for disease control.

Animal health is fundamental for livestock production, welfare, and the safety of food products. Problems associated with animal health have been estimated to reduce the total turnover in the developed world by 17% and production in the developing world by 30–35% [1]. Many animal diseases pose a risk to humans, both consumers, through contamination of the food chain and producers by direct transmission from infected stock. Disease control depends on the accuracy and timeliness of diagnosis, as well as on farm management practices, including nutrition, housing, and breeding. Eradication of infectious diseases is difficult. A new opportunity is presented by the application of the latest technologies and selective breeding approaches to improve disease resistance (Figure 1). This objective will be aided by using omics for improved diagnosis of specific infection-based targets or markers to assist selection. This review will focus on Johne’s disease (JD) in sheep and goats, a disease caused by *Mycobacterium avium subspecies paratuberculosis*, and will consider omics approaches for its diagnosis.

## 2. Johne’s Disease All Over the World

Paratuberculosis, commonly known as Johne’s disease (JD) [2], is a form of chronic granulomatous enteritis caused by *Mycobacterium avium subspecies paratuberculosis* (MAP). The disease predominantly affects ruminants, including cattle, sheep, goats, and many wildlife ruminant species such as deer, antelope, bison, and camels, but has also been found in non-ruminant species such as hares [3], rabbits, and foxes [4]. The causative agent (MAP) is found worldwide [5]. Herd-prevalence is almost 68% of dairy cattle herds in North America [6]. In Europe, there is no accurate data on infection rates in cattle and estimates depend on the test used and the sample size, but it is estimated that more than 20% of dairy herds are infected [7]. In the United States, within infected dairy herds, the prevalence has been estimated at 5.5% of cows clinically affected [6]. There have been a limited number of epidemiological studies in sheep and goats, although a detailed study of JD incidence in sheep was recently conducted in South America, which estimated a within-flock prevalence lower than 2.3% [8]. The national prevalence and economic consequences of JD infected flocks vary considerably depending on climatic conditions, soil types, and production systems.

The information available from the World Organization for Animal Health (OIE) for ovine and caprine JD is far from complete for many countries. The OIE has reported clinical JD in Europe, North and South America, Africa, the Middle East, and Australia. However, data are unavailable for some regions. Animal and herd-level disease prevalence can be gleaned from the scientific literature reporting JD infection in different territories and countries; however, the information is dependent on the testing methodology, sampling strategy, definition of the boundaries of the epidemiological area studied, animal management, age of the animals tested, in addition to various other confounding factors. 

The apparent within flock prevalence of ovine JD in Europe has been reported to be around 4% in sheep and goat herds [9], while a study describing MAP infection prevalence of sheep dairy flocks in Italy [10] found almost 73% of flocks were positive, with a mean within flock seroprevalence of 6.3% when animals were tested by enzyme-linked immunosorbent assay (ELISA). A more recent study in the Apulia region of southern Italy found a seroprevalence of 66.2% for flocks based on a two-year survey of 419 farms [11]. In Spain, the seroprevalence of JD among flocks has been reported to be about 30% [12,13], with a prevalence within flocks of 2–5% [12]. Ovine JD was first reported in sheep in Australia in 1980 [14] in New South Wales and was then reported in other Australian territories. In 2000 it was estimated that JD prevalence was between 6–10% in flocks in New South Wales and that 2.4% to 4.4% of flocks across Australia were likely to be infected [15]. In New Zealand, MAP infection was estimated to be 76% among flocks, using a novel Bayesian method that combined results of two tests, pooled fecal culture, and individual ELISA tests on serum [16]. In South Africa, studies on a large number of animals showed a flock-level prevalence of about 3% [17]. In Latin America and Caribbean countries, JD prevalence at the animal level is estimated at about 16% using a random-effects model analysis of published data [18]. The same study estimated the prevalence of JD in goats to be 4.3% at the animal level and about 3.7% at the herd level, but the heterogeneity among studies is very high.

JD in goats shows similar distribution to that of sheep, although there has been considerably less monitoring of goat herds than sheep flocks. It is difficult to estimate the prevalence of the disease because goats are kept in small groups and generally managed extensively [9]. In a Greek study [19], the prevalence in dairy goats was estimated at about 24.1% between herds and about 10% within-herd. In France, the prevalence of JD in dairy goat herds was reported to be 62.9%, in serologically positive herds, and the average estimate of within-herd prevalence was 11.1% [20]. In a study that examined raw goat’s milk for MAP in Norway, 7.1% of the samples were positive, using an immuno-magnetic separation method and PCR. A similar study was carried out in Switzerland to estimate the presence of MAP in goat bulk milk, which showed that 23% of samples were positive [21]. However, as testing of an adequate number of flocks is rarely performed, the reliability of the information in the literature for within-herd prevalence estimates is low.

These data highlight the wide distribution of JD in small ruminants across the world. MAP causes economic losses for the livestock industry because of decreased milk production, reduced slaughter weight, diminished product value and increased culling rates [22]. Additional economic costs include diagnostic tests, veterinary costs, control measures, and accelerated replacement rates. Losses also occur due to the reduced reproductive efficiency and increased susceptibility to other diseases, although these are difficult to quantify due to the lack of reliable information [6].

As the disease has a long incubation period, many infected animals are in the sub-clinical phase, and prevalence cannot be accurately determined; hence, estimates of economic losses associated with JD are difficult to evaluate. Conservative estimates suggest the combined costs to producers, including losses and increased costs, range from 100 to 200 euro/animal for cattle and from 60 to 120 euro/animal for meat and dairy sheep [23,24]. In Australia, decreased farm gross margin has been estimated to range from 2.2% to 15.4%, depending on the flock prevalence of MAP. Losses due to JD in New Zealand are responsible for two-thirds of the total financial losses associated with sheep deaths [25].

Various strategies to reduce the frequency of Johne’s disease have been used in several countries over recent years. These strategies depend on the livestock species, production system, and the prevalence of JD. National control programs have been established in 22 countries, including Australia, Norway, Iceland, Japan, The Netherlands, Denmark, Canada, and the USA in dairy cattle herds [7]. In Australia and New Zealand, there are national programs for sheep, where this species represents an important livestock resource, as well as in other countries since 2012 [7]. In Norway, Iceland, and in other nine countries worldwide, there is a national control program for Johne’s disease for goats [7,26].

## 3. Transmission and Signs 

Infection with MAP occurs most commonly via the fecal-oral route. Transmission in utero and via milk or colostrum has been reported in cattle and may also occur in other ruminant species [27]. Although MAP infection takes place during the first months of life, visible symptoms rarely appear before two years and usually much later. The age of the onset of the clinical disease tends to be younger in sheep, goats, llamas, and deer than in cattle. MAP is shed in the feces before evident clinical signs develop, infecting other individuals. The long incubation period before the appearance of clinical signs makes the disease very difficult to control, and eradication of the disease from infected herds particularly problematic. Some infected animals may never develop the clinical disease, and whether such animals can transmit the disease is not known. Infection of adult sheep has been seen in experimental conditions but whether this occurs in the field is unclear [28]. 

Goats show a higher susceptibility to infection and develop clinical JD earlier than sheep and cattle [29]. Goats also tend to show a stronger immune response to MAP infection than sheep. There is no evidence for differences in susceptibility among breeds of sheep or goats [30,31], and differences in infection rates among flocks are more likely to be related to differences in farm management systems or production stress between milk or meat-type breeds. 

When the signs of the disease appear, infected sheep and goats show weight loss, decreased milk production, as well as hypo-fertility, and mild anemia. In cattle, intractable chronic diarrhea occurs, leading to severe debilitation, systemic dehydration, and eventually to death in the late clinical phase [2,32]. In sheep and goats, chronic weight loss is also the primary clinical sign of Johne’s disease; however, only 10–20% of clinical cases develop chronic diarrhea in the end stages of the disease [33]. Other visible signs of JD in sheep are hypoproteinemia, intermandibular edema (bottle jaw), fragile wool, and a pre-disposition to increased parasite burden [34]. The end stages of the disease in goats are associated with anorexia, depression, and clumping of faeces [35]. In both species, there is a visible and variable thickening of the distal small intestine, an enlargement of the ileum associated with macroscopic lesions, and edema of the associated mesenteric lymph nodes, with occasional loss of mesenteric fat [36,37,38]. The thickening and lesions of the intestinal tract are found in the jejunum, and necrotic lesions occasionally occur [37,39]. Recently, Wood et al. [40] carried out a serum lipid omics study of control and MAP-infected cattle and found altered availability of choline-containing lipids in the late disease process, probably because of malnutrition and altered biosynthetic capacities of the liver and gastrointestinal tract. Alterations in the bioavailability of these critical structural lipids contribute to the loss of condition of infected animals.

In sheep, three different forms of the disease have been described: paucibacillary, multibacillary, and asymptomatic [36,41]. Only 30% of animals exhibit clinical signs of JD [41] even when the infection is present in the flock. Of the clinically affected animals, 30% develop the paucibacillary form of the disease and 70% the multibacillary disease, the difference depending on the number and ratio of lymphocytes vs. macrophages and the number of bacteria found in the lesions [36]. Asymptomatic animals may be positive for MAP by PCR analysis of faeces but do not show either clinical or histological signs of the disease [41]. This is different from the situation in cattle, where paucibacillary lesions are often present in the early stage of the disease. Sheep with multibacillary JD shed more MAP into the environment than animals with the paucibacillary form of the disease [42]. When replication of the bacteria in macrophages increases, apoptosis occurs, leading to the release of MAP into the lumen of the gut [43], which is then excreted in the faeces.

The main barrier to JD eradication is the lack of diagnostic tools, especially the ability to identify infected animals in the sub-clinical phase of the disease. Currently, JD is diagnosed using either serological or cellular response tests that identify animals once they have mounted a humoral immune response, which does not occur until the later stages of the disease. The direct diagnosis and confirmation of the disease in the clinical phase is by the identification of MAP in faeces through culture. Experimental studies and clinical observations on sheep and goats have identified differences in the immune response to MAP infection compared with cattle [44,45,46].

## 4. Mechanism of Infection and Host Response

Mycobacteria, including MAP, are acid-fast gram-positive bacteria that grow slowly. MAP targets the mucosa-associated lymphoid tissues of the host. MAP strains were initially named depending on the species from which they were first isolated, cattle (C) or sheep (S) types, and were classified following molecular analysis, initially by restriction fragment length polymorphism (RFLP) analysis [47]. More precise classification of MAP strains has been carried out by pulsed-field gel electrophoresis (PFGE), which identified three main types: type I (sheep strain), type II (cattle strain), and type III (a sub-type of type I also known as intermediate strain) [48,49]. The S strain is mainly found in sheep [42], but cross-species infections are observed [50]. Peripheral immune response in sheep to infection with C and S strains of MAP differs; lambs infected with S-type strains only showed focal granulomatous lesions, restricted to the lymphoid tissue, and S-type infection is not associated with a detect able peripheral immune response. The S-type of the lesion has been described in experimental paratuberculosis in sheep and also in adult animals in natural cases, leading to the hypothesis that they could be considered as latent lesions present in adult animals infected earlier in life or as initial lesions of recently infected adult individuals [51].

One route that MAP infects the host is via the micro fold cells (M cells) of the ileal Peyer’s patches of neonatal calves [52], goat kids [53], and mice [54]. The M-cells lack lysosomes and hydrolytic enzymes, which allows the bacteria to survive and be transported intact to the underlying immune-competent, sub-epithelial lymphoid tissue [55]. MAP expresses fibronectin attachment protein (FAP) on its cell wall, which allows its opsonization by fibronectin and then uptake by the M cells, which express several β1 integrin receptors on their luminal surface [56,57]. Following MAP uptake, defensins, which are antimicrobial peptides, are activated by the M cells to protect the host. In the first 4 h after experimental infection of cattle with MAP, a transient increase in β-defensin is seen [58]. MAP has also been shown to be taken up by enterocytes in lambs [59] and mice [54].

After the initial invasion via the M cells, MAP is translocated through the mucosal epithelium, then enters and persists in sub-epithelial macrophages, resulting in the initiation of a cellular immune response based on the CD4+ Th1 cell compartment. The uptake of MAP by macrophages involves complement receptors (CR1, CR3, and CR4), immunoglobulin receptors, and scavenger receptors. The pattern of cytokine activation, tumour necrosis factor α (TNFα) or nicotinamide adenine dinucleotide phosphate (NADPH) oxidase, depends on the route by which MAP enters the macrophages [60]. The recognition of pathogen-associated molecular patterns (PAMPs), for example, lipoproteins on the MAP cell surface, by pathogen recognition receptors (PRRs), such as Toll-like receptors (TLRs), activates intracellular signaling pathways which induce cytokine and innate immune responses [60]. Recognition of the PAMPs by TLRs results in phagosomal cell maturation. In humans, mutations in the TLRs have been associated with variations in susceptibility to diverse pathogens, including mycobacterium tuberculosis [61,62], leprosy [63], pneumococcemia malaria [64], and urinary tract infections [65]. Expression of TLRs changes during MAP infection [56], especially TLR2 and TLR4, which are expressed on the cell surface. Increased expression of TLR2 and TLR4 has also been reported in MAP-infected sheep, suggesting that they play a role in the regulation of immune response [66]. In particular, in sheep, TLR2 has been shown to induce an increase of Interleukin (IL)-10 through the MAPK-p38 pathway [67]. IL-10 has anti-inflammatory activity and is expressed to protect the host from damage resulting from a strong cellular immune response.

MAP is able to prevent maturation and acidification of the phagocytic vacuole within macrophages, thus avoiding exposure of the bacteria to the bactericidal effects of lysosomal enzymes and oxygen-derived radicals [68]. MAP is, therefore, able to replicate within the phagosome of the macrophages using host cell resources. When these resources become limited, it is suggested that MAP induces apoptosis and infects neighboring macrophages, which migrate into the local lymphatic tissue, resulting in the spread of bacteria to lymph nodes, including the mesenteric lymph nodes [69,70]. Depending on the stage of infection or virulence of the strain, MAP can also inhibit apoptosis of macrophages reducing the immune response and allowing intracellular replication [71]. These mechanisms mean that MAP can suppress the host immune cell responses. 

Macrophages present MAP to CD4+ T helper-1 (Th1) cells during the early stages of infection. CD4+ T-cells are capable of secreting interferon gamma (IFN-γ), which restricts bacterial multiplication. In cattle, the level of IFN-γ increases in the ileal and cecal lymph nodes during the subclinical stage of JD. Peripheral blood mononuclear cells (PBMCs) of infected animals show an increased expression of IFN-γ if stimulated with MAP in vitro [72]. Several studies suggest that MAP invades and inactivates the macrophages by subverting their ability to react to normal T-cell signaling. After MAP infection, T-cells are forced to respond to extracellular signals through the CD154–CD40 system, favoring an inappropriate Th2-like activity, including expression of IL-10, and failing to activate phagosome acidification that would kill the bacteria [73].

The course from the subclinical to clinical forms of JD is associated with the change from a Th1 to a Th2 type immune response, which is results in the initiation of a strong humoral response [41] and a simultaneous antibody and IFN-γ response [74]. This mixed cellular and the humoral response has been seen in lambs experimentally infected with a C strain of MAP, whereas animals infected with an S strain only develop a mild immunological response [51]. The immune response of sheep to MAP infection is related to the strain, which affects the type of clinical disease they develop. During paucibacillary enteritis, a cell-mediate immune response of Th1 cells, γδ T-cells, and a ratio >1 of CD4+/CD8+ has been observed [75,76]. Animals with the paucibacillary form of the disease have higher levels of INF-γ [75], whereas sheep that present the multibacillary form show a strong humoral response and a poor cell-mediated immune response to MAP [77] with a lower ratio of CD4+/CD8+ cells. In this latter form of the disease, IFN-γ production is lower [41].

In goats, IFN-γ is the major cytokine involved in macrophage activation and immune response to MAP, at least in the early stages of the disease following infection [78]. In both sheep and goats, as the clinical disease progresses, there is a reduction of the immune response, probably due to an increased expression of the immune-suppressive cytokines [79]. In goats, neutralization of IL-10 in vitro, using monoclonal antibodies, induces the release of IFN-γ from T-cells [80]. This confirms the inhibitory effect of IL-10. In sheep, an increase of IL-10 was observed 4 months after experimental infection with MAP [67], suggesting that in the early stages of the diseased sheep preferentially produce a Th1 immune response over a Th2 immune response. The role of IL-10 in response to MAP is therefore controversial, and the reason for the difference in response between the two forms is still unknown [67]. 

## 5. Importance of Biomarkers

The drawbacks of diagnostic tests commonly used to detect JD infection are that they do not have adequate sensitivity or specificity and invariably detect the disease in the later stages when the clinical signs are already present. Tests for MAP antibodies using enzyme-linked immunosorbent assays (ELISA) or agar gel immunodiffusion (AGID) are able to detect the humoral response to MAP but are only effective in the late stage of infection. This is confounded by differences among animals in their manifestation of infection and between strains. Differences between the S-type sheep strains and C-type cattle strains [81] may be reflected in differences in immune response between the two species and hence differences in the accuracy of indirect tests such as the ELISA test [82]. Trials in Australia and New Zealand have shown that for small ruminants, the AGID test has a slightly higher sensitivity and specificity than the ELISA [83,84,85]. The specificity and sensitivity of the AGID test in comparison with the histological determination of MAP infection were 99–100% (95% CI) and 38–56% (95% CI), respectively [85]. To diagnose infection at an earlier stage, detection of IFN-γ produced by white blood cells specifically stimulated with MAP antigen lacks specificity (about 77% in cattle) [86]. Whereas the identification of MAP in faeces by PCR assay can produce false-positive results, firstly because of the cross-reactivity of the PCR target sequence, IS900, with other mycobacteria such as *M. cookie* and *M. scrofulaceum* [87], and also because some animals can ingest MAP from the environment and excrete it in their faeces without being infected, although this is less of a problem for sheep and goats than for cattle [88]. It should be noted that MAP is generally not present in the faeces of animals in the early stages of the infection. New diagnostic tests are required to diagnose JD in the early preclinical phase to support disease eradication programs and reduce the spread of the bacteria. Ideally, these tests should be based on biomarkers that are easily accessible and can be tested on samples obtained using a minimally invasive technique. Hence biomarkers that can be assayed in body fluids, such as blood, milk, or urine, are particularly attractive. Such biomarkers have been developed for human medicine to diagnose heritable and infectious diseases. The host transcriptome and/or proteome, for example, may provide opportunities to improve the diagnosis of JD during the subclinical stage of the disease when both direct and indirect detection of infection is ineffective.

## 6. Biomarker Discovery by Transcriptomic Approaches

Gene expression profiling is widely used to study both metabolic, oncologic, and infectious diseases in humans. Identifying a specific transcriptional signature related to the host immune response in the early stage of infection could facilitate a precocious diagnosis. Transcriptomic profiling has been used to differentiate patients infected with Dengue hemorrhagic fever that will develop the more severe form from those that will develop milder signs of the disease [89].

Several transcriptomic studies of JD in farm animals have focused on the mechanisms of entry and survival of MAP in macrophages [71,90,91,92,93,94]. Although conditions and experimental settings differ between studies, pathway analysis identified that lipid metabolism, immune response, and antigen presentation were usually involved. Cholesterol accumulation and modification of lipid metabolism after MAP infection were identified in more than one study [91,95]. Johanesen et al. found an accumulation of cholesterol in MAP infected murine macrophages, indicating a mechanism of MAP to survive in host cells, although the process of activation of genes involved in lipid endocytosis and synthesis appear strain specific. The authors suggest that these mechanisms could be shared between bovine and ovine macrophages, although this has to be confirmed. Other studies have examined changes in Th1 cell receptors [66,96], differential expression of proteins that regulate apoptosis [71], and variation in cytokine mRNA expression [97], both to better understand the pathogenesis of the disease and to find potential biomarkers to distinguish the immunological state of the host. 

In cattle, a 10-gene expression signature (*TRPV4*, *RIC8B*, *IL5RA*, *ERF*, *CDC40*, *RDM1*, *EPHX1*, *STAU1*, *TLE1*, and *ASB8*) has been described that is able to discriminate between ELISA-negative, clinically healthy and JD exposed animals [98]. This finding suggests that RNA expression analysis may be useful as a diagnostic tool to identify infected or exposed animals before they become seropositive, which can be detected with the ELISA test. In sheep, gene expression patterns in tissues such as the terminal ileum, terminal jejunum, and mesenteric lymph nodes depend on the pathological form of JD [41,66,97,99,100]. These studies have focused on the differential expression of pathogen recognition receptors (PRRs) as biomarkers, given their pivotal role in initiating an immune response to pathogens, including MAP.

Studies in vivo and in vitro have reported changes in toll-like receptor (TLR) gene expression in sheep during experimental infection with MAP [66,96,99,100]. TLR2 is involved in regulating host immune response in the early stages of JD and affects the progression of the disease, both in target tissues [66,100] and in PBMCs [96] of sheep. In the multibacillary form of JD, TLR2 mRNA expression increases [66,96,100], probably due to the greater inflammatory response in the lesions where most bacteria are found. Expression of TLR4 is also increased in animals with later stages of the multibacillary form of the disease [96,100]. Whereas in cattle PBMCs, TLR2 expression decreases following MAP infection, and TLR4 shows no significant difference in expression [96]. Differences in immune response related to the MAP S or C strains may affect the course of the disease. Expression of TLR1 and TLR 6 [99], in particular the expression of TLR6 in sheep, increases in target tissues in both paucibacillary and multibacillary forms of the disease. The increase of expression of both TLR6 and TLR2 enables the TLR2/TLR6 heterodimer to form in response to MAP [99]. In addition, transcription of TLR9 increases more than 10-fold in dendritic cells and target tissues in sheep naturally infected with JD, even during the asymptomatic phase of the disease [100]. Increased expression of TLR9 is also seen in PBMC of cattle experimentally infected with MAP [101]. However, other studies have reported that no changes in TLR9 expression are found in PBMCs of sheep and cattle naturally infected with MAP [96]. Activation of TLRs associated with mycobacterial infection, especially in macrophages and dendritic cells, leads to the stimulation of intracellular signaling pathways that in turn result in the production of proinflammatory cytokines (i.e., TNF, IL-6, and IL-1) through MAPK and NF-κB pathways [102,103]. These expression signatures have been used to explored MAP infection of sheep and goats [66,97,100,104]. In cattle IFN-γ is used in current diagnostic tests as a marker of exposure to MAP; however, it is not a good marker of infection due to its low specificity [105].

The expression of cytokine genes and increased expression of IFN-γ, TRAF-1, IL-8, and TNF-α reflect the inflammatory status of the target tissue [41]. A significant difference in TNF-α and IL-18 expression may allow the differentiation between asymptomatic infected animals and controls [41]. Differences between multibacillary and paucibacillary JD are also reflected in IL-10 expression, which is higher in the multibacillary form compared with the paucibacillary form, whereas in the latter, there is a higher expression of IFN-γ and TRAF-1 [41]. In sheep, goat, and calf infection models where animals received 109 CFU of an isolate of MAP in milk replacer, a marked antigen-specific IFN-γ response was seen in sheep 90 days post-infection [106], whereas it took calves 360 days, to show an increased response to IFN-γ. In this model, the relative gene expression of *IL-4*, *IL-12*, and *IL-17* in peripheral blood mononuclear cells (PBMCs) was higher in goats than sheep, which corresponded to the lower tissue colonization. These results show that there are discrete differences in host responses between species.

Differences in cytokine mRNA expression are seen among naturally infected animals, which may reflect the uncertainty in the time and the level of infection following natural exposure. Different levels of expression of immune-inflammatory genes are seen between the different forms of ovine JD. Eight genes, *CD63*, *CXCR4*, *IGFBP6*, *IGF2R*, *ITGB2*, *MMP-9*, *TLR2*, and *TYROBP*, are involved in the inflammatory pathways related to mycobacterial disease and show different levels of expression depending on the form of the disease [97]. *IGFBP6* has an increased level of expression in sheep with the paucibacillary form and also shows increased expression in cattle PBMC stimulated with MAP [72]. *TLR2* is over-expressed in the paucibacillary form of JD in sheep [97]. IL23, a regulator of inflammation at the mucosal surface, is produced by macrophages, and dendritic cells and is over-expressed in the ileal mucosa of sheep with a paucibacillary form of JD [107]. This is probably due to the higher level of macrophage infiltration in the paucibacillary form compared with multibacillary lesions. These data suggest that while the expression of individual genes cannot be used as a biomarker for infection, the expression signature of a carefully selected panel of genes may be diagnostic of MAP infection, even during early stages.

IFN-γ induces IDO (Indoleamine-pyrrole 2,3-dioxygenase) expression and its activity is considered a prognostic factor in human patients with pulmonary TB [108]. IDO is an enzyme that regulates tryptophan metabolism, but it also shows an immune-regulatory activity following microbial infection. IDO mRNA levels are significantly increased in target tissues (ileum, jejunum, and ileum draining lymph nodes) of Merino sheep experimentally infected with JD compared with controls [109]. Sheep with the multibacillary form of JD shows the highest expression level of this enzyme, suggesting a correlation between IDO expression and the severity of the lesions. PBMCs, both from naturally infected and experimentally infected sheep, also over express IDO. Immunohistology has shown an increase of IDO protein in the gut of infected sheep. Subclinical MAP infection reduces the expression of immune regulatory genes, *IL-17A*, *IL-17F*, *IL-22*, *IL-26*, *HMGB1*, and *IRF4* in cattle blood and increases expression of *PIP5K1C*, which is consistent with the suppression of the Th1 response [110]. Furthermore, increased expression of *IRF5* and *IRF7* is seen, suggesting that IFN-α/β signaling is activated during subclinical stages of the disease. This would be induced by a reduction in tryptophan metabolism, which would be a direct consequence of the indoleamine 2,3-dioxygenase function [110]. 

Most studies of gene expression performed in vitro have been on bovine macrophages infected with MAP because of the role of this cell lineage in mycobacterial infection and T-cell activation. Studies of the expression of cytokines have reported different responses between cattle and small ruminants. Barbari goats in India showed an increased expression of *IL2* and *IFN-*γ (3.93-fold and 9.6-fold, respectively) four months post-infection (MPI) versus controls. However, at eight MPI, expression of *IL2* declined to below control levels, whereas *IFN-*γ mRNA expression was consistently increased during the entire experimental trial, at 4, 8, and 12 months in infected goats [111]. *IL10* expression was also elevated at 8 and 12 MPI [112]. 

The insertion element IS900 of MAP has been detected in the blood of experimentally infected Jamunapri and Barbari goats by PCR [113], with a positive result in 77% of the 111 animals tested, both adults and kids. Detection of the IS900 sequences by PCR was also tested as a JD diagnostic in Australian sheep [83], but only 2 of 14 experimentally infected animals were positive to histology and PCR for MAP on samples of ileum and ileocecal lymph nodes at necropsy. 

## 7. miRNA as Biomarkers

Changes in the levels of microRNAs (miRNAs), which regulate gene expression, have also been examined as possible biomarkers of infection [114]. MicroRNAs are small non-coding RNA molecules that regulate a variety of cellular processes, including differentiation, cell cycle, and apoptosis [115,116], and hence play a fundamental physiological role. Therefore, miRNAs are potential candidates as biomarkers for physiological and disease status [117]. Examining miRNA expression may help to better understand how the host responds to disease challenges. The study of miRNAs expression in farm animals has initially focused on their role in regulating production-related traits such as muscle development and hypertrophy, adipose tissue growth, or fertility [118].

More than 200 miRNAs have been identified from ovarian follicles and corpus lutea of Blue Faced Leicester and Scottish Blackface cross sheep breeds [119]. The expression of 159 miRNAs has been compared in Texel and Ujumq sheep to identify the difference between these two breeds in wool production [120]. MiRNAs have been shown to be involved in muscle development in Huanghuai goats, a Chinese meat-producing breed [121], and also in hair cycle [122], hair follicle growth, and development [123].

In addition, miRNA expression has been shown to regulate physiological and pathological processes [118,124] in mice [125], cattle [126], and pigs [127]. MiRNA expression of both the host and pathogen can affect the course of disease [128]. MiRNA expression profiles have been shown to change based on cancer tumor type and status. MiRNAs have been used in the diagnosis of cancer and its prognosis and to guide the choice of treatment during tumorigenesis in humans. MiRNAs regulate the host immune response to pathogens, e.g. they affect the development, differentiation, survival, and function of B- and T-lymphocytes, dendritic cells, macrophages, and other immune cell types [129].

Toll-like receptor signaling is regulated by miR-155, miR-21, and miR-146 [130], antigen presentation by miR-155 [131,132], cytokine responses by miR-146, and T-cell receptor signaling by miR-181a [133]. MiRNAs have been implicated in viral immune escape and viral defense [134]. MiRNAs are also involved in mechanisms of signaling and defense of the host against a range of microorganisms [135].

In cattle, miRNAs expression has been studied in alveolar macrophages. MiR-21, which is the most highly expressed miRNA [136] in these cells, is involved in the regulation of inflammatory response and has been found to be up-regulated in human monocytes infected with *M. leprae* [137]. This suggests that this miRNA plays a role in reducing host defense, allowing replication of the invading bacterium. Recently, Gupta et al. [138] identified four miRNAs (miR-1976, miR-873-3p, miR-520f-3p, and miR-126-3p) that, in combination, have the potential to distinguish healthy animals from severely MAP infected animals and that may possibly be used to detect MAP infection in the early stages. Although this is preliminary data, such a small set of four miRNAs could offer an easy and cost-effective real-time PCR-based test for JD diagnosis.

Comparison of MAP-challenged calves with age-matched controls by RNA sequencing showed no significant differences in miRNA expression between the two groups 6 months after infection [139]. However, the level of miR-205 increases, and miR-432 decreases with age and developmental stage, which will confound the development of a miRNA-based diagnostic. Assessing gene expression and circulating miRNA in cows positive, exposed, and negative for MAP showed that specific pattens of expression were associated with status [140]. The authors identified differences in the levels of miRNAs, interestingly five were reduced, and three miRNAs increased in the exposed compared to the unexposed group. The miRNA levels were negatively correlated with the expression of their respective target genes, which are known to be involved in the immune response. These data suggest that miRNA profiles may be used to identify infected animals during the sub-clinical phase, which would represent an important advance in preventing pathogen diffusion and reducing transmission. 

Monitoring experimentally MAP exposed and unexposed Merino sheep over a long time period by RNA sequencing enabled expression patterns to predict disease outcome to be identified [141]. Many of the differentially expressed genes identified were involved in immune response, including the MHC class I and class II and T-cell receptor genes. RNA-sequencing of peripheral blood mononuclear cells (PBMCs) from infected, vaccinated, and control goats during early stages of infection identified several differentially expressed (DE) genes between the three different groups, of which many were involved in immune response including *IL-18BP*, *IFN-γ*, *IL-17A*, *NOS2*, *LIPG*, and *IL-22* [142]. 

The rapid improvements in RNA-sequencing have led to the discovery of expression patterns that may provide the basis of diagnostic tests for mycobacterial infections at an early stage [143]. 

## 8. Biomarker Discovery by Proteomic Approaches

Many standard diagnostic methods, such as hematology and serum chemistry/immunology, use blood as a convenient and accessible sample. Physiological and pathological processes modify the composition and abundance of thousands of proteins in serum, and each process may generate a unique protein pattern signature [144]. In veterinary medicine, serum protein analysis has been used to evaluate the clinical status and pathogenesis of diseases for several decades; however, approaches available have been limited to the measurement of the total protein level or specific immunoglobulins and proteins. It is now possible to analyze the overall distribution and abundance of proteins in a mixture using mass-spectroscopy approaches, which can even identify less abundant proteins.

Serum proteins such as signaling factors or molecules released into the plasma from different body tissues are a potential source of biomarkers [145]. The major difficulty when working with serum is the presence of high abundance proteins, such as albumin, that mask those in low concentration; 10 proteins account for 90% of the protein in serum, while the remaining 10% is composed of proteins that show a wide dynamic range, differing by more than 10 orders of magnitude in concentration [146]. Therefore, pre-analytical enrichment is crucial to reduce the high abundance of proteins. Immuno-depletion (ID) or antibody-based depletion can be used to remove high abundance proteins or to enrich the low abundance protein fraction. 

Mass spectrometry (MS)-based proteomic analysis has identified several serum biomarkers in sheep and goats [147,148]. Surface-enhanced laser desorption/ionization time-of-flight mass spectrometry (SELDI-TOF MS) has been used to find serum biomarkers for Fasciola hepatica infection in sheep. Starting from the analysis of more than two thousand (2302) protein clusters that varied in intensity during the 12 weeks post-infection, 26 proteins were identified as biomarkers diagnostic of disease status [149]. SELDI-TOF MS proteomic profiling of sheep serum has also been used to detect molecules related to the immunological response to foot rot vaccination [150] that led to the identification of four putative biomarkers.

The investigation of proteins present in the serum of MAP-infected animals could increase knowledge of the pathogenic processes associated with mycobacterial diseases. This information may lead to the development of MAP-specific diagnostic tools to monitor the progression of the disease. SELDI-TOF MS has been used to analyze the proteome in serum from Merino sheep naturally infected with JD and vaccinated sheep at three timepoints (4–8 and 13 months) after infection and uninfected controls in a longitudinal study over 13 months [151]. Several differences in the protein profile between infected (*n* = 30), vaccinated (*n* = 30), and unexposed control animals (*n* = 29) were observed. A large number of proteins were found to be in common among infected and vaccinated animals, compared with controls, many of which were associated with the immunological response [151]. In particular, alpha-hemoglobin and transthyretin (TTR) were identified as candidates for serum protein biomarkers of JD disease. Proteomic studies have shown that TTR is significantly reduced in JD infected and vaccinated Merino sheep [151]. TTR, a 55 kDa carrier protein, is known to transport thyroxine and tri-iodothyronine, as well as vitamin A (retinol or trans-retinoic acid) through association with the retinol-binding protein in serum in humans [152].

Conversely, studies in cattle found an increase of TTR in animals experimentally infected with MAP [153]. TTR has been suggested as a biomarker for tuberculosis in humans from a study evaluating serum proteomic profiles of patients with active tuberculosis and controls [154]. Investigation of serum proteome variations in response to *M. bovis* and *M. paratuberculosis* using iTRAQ and liquid chromatography coupled with mass spectrometry has identified protein expression patterns that can differentiate between the biologically related mycobacterial diseases in cattle [153]. Transthyretin, a retinol-binding protein [152], and cathelicidin have been identified exclusively in *M. paratuberculosis* infection, while increased serum levels of alpha-1-microglobulin/bikunin precursor (AMBP) protein, alpha-1 acid glycoprotein, fetuin, and alpha-1B glycoprotein were detected only in *M. bovis* infected animals [153].

Retinoic acid, transported by TTR, is involved in monocyte activation and inhibition of M. tuberculosis multiplication in human macrophages [155]. Retinoic acid is involved in the inhibition of in vivo growth of *M. tuberculosis* and in the development of tuberculosis in experimentally infected rats [156].

A study in cattle identified potential biomarkers of JD from plasma of JD infected Holstein cows by 2-dimensional Fluorescence Difference Gel Electrophoresis (2D-DIGE) [157]. Six proteins differed between MAP positive and MAP negative animals; four had an increased level in infected animals (Transferrin, α/β gelsolin, complement subcomponent C1r, C3, amine-oxidase copper containing 3, and thrombin), and the level of two decreased (Coagulation factor XIII and fibrinogen ϒ chain). Transferrin, a hepatic iron-binding protein, was found to be over-expressed in cows with chronic infection. This may be explained by the need for a better uptake of iron which is known to be reduced in infected animals due to the damage of intestinal mucosa [158]. Transferrin over expression in serum of infected cattle was validated by ELISA test using specific antibodies [157].

Alteration of the level of the clotting factor has also been associated with JD infection in cattle [158]. This protein reduces bleeding and microbial invasion, and increased expression has also been seen in inflammatory bowel disease (IBD) patients where there is an increased risk of thrombosis [159].

Immunoreactive proteins in the serum of cattle have been studied by proteomic approaches to better identify JD-infected animals, improve the efficacy of the available ELISA tests and explore the antibody response to the MAP vaccine [160]. Six immunoreactive proteins have been shown to be differentially expressed between MAP infected vs. control cows by 2D/ MALDI TOF analysis of serum, namely the 65K heat shock protein (Hsp 65), malate dehydrogenase, an uncharacterized oxidoreductase MAP_3007, a putative protein MAP1386c, a Major membrane protein-1 (MAP2121c,) and Fructose 1.6 bisphosphate aldolase [160]. Hsp 65 causes a strong immune response in experimental and natural JD infection. Mice infected with *M. avium* produce antibodies that react with recombinant Hsp65 of MAP [161]. Interestingly the protein shares amino acid sequences with other human proteins such as superoxide dismutase [162]. Hsp65 is involved in systemic lupus erythematosus, chronic active hepatitis, and atherosclerosis [163], suggesting its role in an autoimmune response in human diseases that are related to MAP, such as multiple sclerosis and Type 1 diabetes [164].

Further, in vitro proteomic analysis of macrophages at different stages of infection with MAP has been recently conducted by mass spectrometric approaches [165]. The aim was to gain knowledge on the host response and signaling processes that allow bacterial persistence and spread within the bovine host. The results showed that the passage of MAP through bovine epithelial cells increases the synthesis of integrins allowing a more efficient translocation of the bacterium. These findings confirm the role of integrins in MAP migration and in the pathogenesis of infection [57].

A proteomic study in sheep using a shotgun proteomics approach based on mass spectrometry of ileal tissues of JD infected ewes and controls identified 2889 proteins. Of these, 384 showed differential expression between ewes with or without JD infection, of which 341 had a higher expression in JD ewes [166]. The bioinformatic analysis placed these proteins in pathways affecting inhibition of phagosome acidification, bacterial invasion, leucocyte recruitment and activation, and antimicrobial activity [167]. The same authors studied the paucibacillary state versus control status in sheep combining shotgun proteomics, histopathology, and immunohistochemistry of ileal tissue and identified 96 differentially expressed proteins, mostly involved in immune response and in macrophage-MAP interaction.

## 9. Conclusions

Currently, there are no reliable methods to identify JD-infected animals in the early stages of the disease. This makes it difficult to develop efficient control strategies for the eradication of the disease. The development of efficient diagnostic systems for the identification of MAP infection in preclinical animals is a research priority. The discovery of specific microRNA and serum protein signatures associated with the early stages of the disease may lead to new tools for the early diagnosis of MAP infection in a simple, accurate, and non-invasive assay. Innovative tools for both diagnostic and prognostic purposes for veterinarians and breeders would have significant outcomes for both animal and public health by facilitating cost-effective monitoring and the control of the disease. 

Several projects have addressed the identification of markers of early infection, especially in cattle. Early biomarkers are essential to develop effective control strategies and may also contribute to the development of more effective vaccines. The opportunities for identifying biomarkers have increased with the sequencing of sheep and goat genomes and improvements in transcriptome and proteome profiling techniques. As a result, the number of putative JD biomarkers has increased dramatically. Future research in this field will characterize the effective immunological response to this disease and the genetics of variations in susceptibility. Genomic-based selection for resistance would help breeders to reduce disease incidence and the economic losses due to Johne’s Disease.

## Data Availability

Not applicable.
